# Assessing family relationships through drawing: the Family Life Space

**DOI:** 10.3389/fpsyg.2024.1347381

**Published:** 2024-08-05

**Authors:** Marialuisa Gennari, Caterina F. Gozzoli, Giancarlo Tamanza

**Affiliations:** Psychology Department, Catholic University of Sacred Heart, Milan, Italy

**Keywords:** family relations quality, family dynamics, family clinical intervention, family assessment, interactive tool, relational clinical tool

## Abstract

The Family Life Space (FLS) is a graphic instrument that may aid the relational assessment of families. This interactive instrument involves all members of the family in a joint task, that of collectively making a drawing of their own family system and it allows the gathering of information related to the overall family organization. The FLS was originally conceived by Danuta Mostwin in the early 70s and used as an instrument for clinical intervention. After having been applied to several contexts, the present contribution aims at presenting the key indicators to use the instrument as a tool for assessing family relations. Specifically, the characteristics of the instrument that allow the gathering of relevant information at the individual, relational, and family level will be outlined. For each of these levels, the data regarding the quantity and quality of the elements in the drawing that define the family space (i.e., the number and graphic quality of the actual elements in the drawing) as well the quantity and quality of the relationships among family members and with their community at large (i.e., the number and type of lines connecting the various elements in the drawing) will be presented. The instrument can therefore provide useful insights on the following constructs: quality of life, power dynamics within the family, feelings of belonging, closeness and/or distance as well as conflict or acknowledgement between family members and the overall attitude family members have toward their context and the critical events they had to face. The application and complete potential of the instrument are further elaborated upon through the presentation of a clinical case. This case not only aids in comprehending the tool’s usage but also enables the collection of psychological information about the family and provides a clinical interpretation of family relationships.

## 1 Introduction

The comprehensive understanding of family functioning, beyond a mere description of its interactive patterns, represents the ultimate objective of a relationally-oriented family assessment. Specifically, employing a relational lens in family assessment involves delving into the meanings and motives underlying family actions, thereby grasping the “whys” of observed behaviors and narratives, not just the “hows.”

This process demands not only a specific theoretical orientation but also a consistent set of tools and procedures. To attain this objective, both the setting and the entire data collection process must be conducive to gathering relational information—data that transcends individual family members and instead weaves together the various pieces of information produced by different individuals within the family.

The Family Life Space (FLS) emerges as a graphic-symbolic tool particularly well-suited for use in a relationally-oriented family assessment procedure. This interactive instrument engages all family members in a collective task, facilitating the collection of information regarding the overall family organization. Initially conceived as a therapeutic tool by Danuta Mostwin in the early 1970s, in the international context its utilization and scholarly examination have been infrequent (Barker et al., 1997; Beeton and Clark, 2019) Introduced in Italy in 1978 (Cigoli and Galbusera Colombo, 1980), the FLS has been utilized in various studies by researchers at the Center for Family Studies and Research in Milan. Its diverse adaptations and applications underscore its flexibility and versatility, expanding its application contexts (Gennari et al., 2015). Twenty-five years ago, the FLS underwent further modifications, especially concerning data gathering and interpretation.

Gozzoli and Tamanza (1998) developed a coding system providing a metric analysis of the graphic-symbolic products obtained through the administration of the FLS. The authors devised an algorithm capable of transforming the symbolic family drawing into a series of mathematical and geometric indicators, considering both its individual elements (lines, points, space occupation density, etc.) and the overall composition (the gestalt formed by the collection of all the individual elements) (Tamanza, 2018).

This article will briefly introduce the tool and discuss its methodological characteristics and assets, with a particular focus on its role as a clinical instrument for family assessment.

## 2 The tool’s theoretical foundations

The theoretical underpinnings of the Family Life Space (FLS) draw upon a comprehensive conceptual framework that encompasses the “ecological perspective,” Lewin’s Field Theory, General Systems Theory, and Symbolic Interactionism.

Consistently with Kurt Lewin’s definition of field, Mostwin defined the family life space as a “bio-psychosocial territory characterized by meaning” (Mostwin, 1980). The Family Life Space centers on spatial analysis, presuming the spatial representability of psychic reality (Hartung, 2013; Birdwell-Pheasant and Lawrence-Züniga, 2020; Wahl and Gerstorf, 2020). Put simply, it suggests that the structures and dynamics within each individual, particularly in family relationships, can be represented through graphic symbols.

The graphic outputs of the FLS provide a tangible representation of family organization, reflecting its capacity to be either a welcoming and warm or a hostile and distancing space for its members. The end result goes beyond a mere representation of the family and its relationships; instead, it encapsulates a compilation of representations and experiences that, through the collaborative effort of all family members, convey the meanings and characteristics associated with the family as a whole.

The fundamental postulate is one of homology, not mere analogy, between family actions and their graphic products (Gozzoli and Tamanza, 1998, cit.).

The instrument relies on a theoretical principle asserting that space not only serves as a container for representation—enabling the graphical depiction of the family organization—but also concretely shapes family action. In other words, the space on the paper symbolizes the actual emotional and relational space (or lack thereof) the family provides for its members and their interactions. In this perspective, the FLS is not merely a projective tool, it is deeply imbued with experiential and emotional connotations, serving as a context where family relationships are constructed, organized, and reproduced.

Within this framework, the FLS is a valuable instrument in working with families as it has proven particularly suited to analyze relations and comprehend the functioning of the family as a whole.

## 3 The administration and the rationale of the tool

The following materials are necessary for the Family Life Space (FLS) administration:

- Vertically-oriented white sheet (50 cm by 70 cm) with a 14 cm-radius circle drawn in the center using a black marker.
- Markers of different colors.
- A sheet for the researcher/administrator’s observations.
- A recorder or video recorder to keep track of the family members’ interactions during the administration.

The sheet should be positioned on a wall or any support perpendicular to the floor, allowing family members a shared perspective on the drawing. All family members must stand on the same side of the sheet, ensuring uniformity in orientation (top, bottom, right, and left) for a unified interpretation of the drawing.

The specific administration involves the following steps:

The researcher presents the sheet, explaining: “This circle represents your family space, while the outer space is the environment that surrounds it. Therefore, things, people and whatever you consider as part of your family should be drawn inside the circle or on its border, whereas whatever you view as not being part of your family should be placed outside of the circle.”

Detailed instructions follow:

1. Indicate yourself with a symbol (point or circle) and assign each symbol you draw a progressive number. You should retain the same marker throughout the drawing.
2. Use a symbol (point or circle) to represent other important individuals in your life, such as relatives, friends, or acquaintances.
3. Once again using the same symbols, indicate important life events as well as significant organizations, groups, and institutions.
4. Mark the quality of relationships among family members using three types of lines connecting the symbols among them: a straight line indicates a good relationship, a dotted line indicates a fair, “so-so” relationship whereas an interrupted line indicates a conflictual relationship.

One of the tool’s strengths lies in its simplicity of application and execution. A white sheet with a circle serves as a metaphor for the family space, and family members use symbols to represent their mutual positions and relationships. The drawing, accompanied by verbal and non-verbal communication, becomes valuable material for the family’s relational assessment.

Moreover, the instrument can be administered twice during the same session, inviting the family to imagine their situation at a certain timepoint in the past or in the future, depending on the clinical objectives.

When explaining the tool, family members need to understand the placement of symbols and the representation of emotionally significant events.

These straightforward instructions, albeit somewhat ambiguous, guide the family in addressing the task. Family members are then asked to indicate the perceived quality of relationships using lines, revealing not only the relationship qualities but also whether they are aligned, distinct, or share a common line of action.

## 4 Analysis criteria

The Family Life Space (FLS) has been conceptualized and employed since its development with clinical purposes and mainly used within a qualitative research methodology that relies heavily on inferential procedures of a phenomenological-interpretative nature (Mostwin, 1980). The interpretive process assigns psychological meaning to the graphic elements presented by family members, forming the basis for the evaluation of the depicted elements.

Within this framework, specific emphasis is placed on certain formal and topographic aspects of the representation. These include the frequency, quality (positive or negative), and positioning of individual elements, the presence or absence of lines, the distribution within and outside the circle, and the presence vs. absence of symbols in specific areas of the drawing, specifically the center and the border of the circle. The overall representation, its gestalt, is also considered, and this aspect is associated with an overall assessment of the family functioning.

The main indicators for interpreting the FLS can be summarized as follows. These indicators arise as clinically meaningful given the research and clinical applications of the tool over the past 25 years. The FLS has been utilized across various contexts, including assessment, consultation, and psychotherapy, catering to a range of populations such as families with elderly members, families of adolescents, immigrant families, as well as separated and blended families and family with disabled children (Gozzoli and Tamanza, 1998, cit.; Gozzoli and Tamanza, 2000; Tamanza, 2000; Onnis et al., 2010; Canzi and Rosnati, 2011; Gozzoli et al., 2012; Gennari et al., 2015, cit.; Gennari et al., 2018; Tamanza, 2018). In all scenarios where the FLS has been utilized, the indicators we present hold significance in distinguishing between family dynamics and functioning:

1. Drawn symbols: The coding procedure involves several steps. Initially, the symbols drawn by each individual family member and collectively by the entire family are tallied. Comparing the number of symbols drawn by each member offers valuable insights into specific family dynamics, such as the level of intimacy, willingness to disclose oneself in front of others, and the power and influence of one family member over the others (Olson, 2000; Madanes, 2014). However, the primary focus lies in the qualitative interpretation of the drawn symbols, which provide relevant clinical information. This includes identifying who is included in the drawing and who is omitted, recognizing repetitions of symbols as well as symbols that are unique to a specific family member. Quantifying each participant’s contribution to the overall representation sheds light on how family members allocate roles, responsibilities, tasks, power, and affection. The comprehensive evaluation of symbols facilitates both quantitative assessment, indicating the richness or paucity of family elements and themes, and qualitative assessment, revealing joyful, significant, problematic, and dramatic events the family has encountered.
2. Connecting lines: In this case, it is important to record the quantity of lines connecting the various elements in the drawing. This entails recording both the number of connections drawn by each family member and the overall count of connections. Moreover, the quality of such connections (positive, negative, or neutral) is also acknowledged together with the member(s) responsible for drawing a higher versus lower number of connecting lines. The overall consideration of the number and quality of connecting lines provides an insight on the complexity and nature of family bonds (Szydlik, 2012): connections solely among elements within the family, connections solely with symbols external to the family, or balanced connections between internal and external elements provide a direct understanding of the family enmeshment/detachment and of its inner and outer boundaries (Minuchin, 2018).
3. The center of the circumference holds geometric significance, being equidistant from every point on the circumference. As per the homology principle previously highlighted, occupying the center signifies relevance, power, and centralization of the family organization. It also indicates the presence of significant relations with other elements in the drawing and among family members (Mostwin, 1982).
4. Occupation of the circumference: The border of the FLS’s circle is a defining line, separating and enclosing, creating a space that separates the inside from the outside. In this respect, particular attention should be placed to which symbols fall within the circle and which are placed on the border or outside the circumference. The topic of family boundaries has been extensively explored by Minuchin (2018), cited; who defined families as centripetal or centrifugal, based on their ability or inability to relate to and integrate external elements. In this context, the occupation of the border may also signify the family members’ capability to create and share a liminal space for connection. The border is viewed as a common area bridging inner and outer spaces while also creating a shared ground among family members. In essence, borders are openings that facilitate the encounter with the others and openness to novelties, thus symbolizing a willingness to change and transform (Cigoli, 1992, cited; Gennari and Tamanza, 2022). In this perspective, determining whether only specific family members can cross and inhabit the boundary or whether this represents a distinctive family trait is particularly interesting.
5. Gestalt coding: This aspect allows for an understanding of the family as a unit. When the overall composition is considered, the above-mentioned homology between the drawing-making process and the family organization is most relevant. In fact, it is precisely the gestalt which uncovers the signs of family spatiality, understood not as a geometric space, or a purely representative one, but as a lived space, filled with affections and meaning. The gestalt resulting from the collection of all the individual symbols shows a representation of the family organization and it enables the emergence and understanding of the family dynamics. Over time, the systemic-relational paradigm (Cigoli and Scabini, 2006) has grappled with the challenge of gathering supra-individual information. This instrument permits the observation of family members individually, as well as in dyads (Tamanza et al., 2018) and triads. This is particularly pertinent as the theoretical foundations of the relational-symbolic model posit that relations are better understood from a triangular perspective or using a triangular matrix. Additionally, the overall image is indicative of the “gestalt” (Cigoli, 1992, cited; Lobb and Conte, 2018) - the specific form experienced and expressed by the family at a given moment. The gestalt coding process involves two levels: the first pertains to the analysis of the geometric figure obtained by ideally connecting all the outer symbols drawn by each family member and comparing the resulting polygon with those of other family members. This identifies, for each family member, the portion of space occupied and its relation (closeness vs. distance; up vs. down) to the space occupied by others. The second level involves comparing the area of the circle to the geometric figure obtained by connecting all the outer elements drawn by the entire family. This comparison yields four possible scenarios: (1) “concentration,” where the family’s polygon is contained within the circle but does not occupy the entire area; (2) “filling-saturation,” where the circle is dense with symbols and overlapping connections; (3) “measurement,” where family members each occupy a specific sector of the circle with few close symbols; and (4) “separation,” where family members occupy specific sectors with no contact or shared areas (Gozzoli and Tamanza, 1998, cited; Tamanza, 2018, cited).
6. Comparison between two FLS productions - present/past or present/future: Families can be administered the instrument twice, providing a representation of the family in the present and offering insights into the family’s past or potential future. Depending on the clinician’s goals, family members are instructed to reflect on a specific moment in their past or imagine a moment in the immediate or distant future. The two versions of the drawing are then compared and evaluated based on the aforementioned indicators. This examination allows for an assessment of changes that have occurred or are expected/feared, offering valuable prognostic indicators and insights for clinical work. Such comparisons may also provide insights for future work, revealing shifts and transitions promoted or hindered by the family, deepening our understanding of family functioning (Cigoli, 1992).

Table 1 provides a concise summary of the areas and indicators used in the FLS interpretation.

**Table 1:** Indicators of Family Life Space.

Area of the sheet	Unit of analysis	Unit of observation	Data collection	Clinical insight
Points/symbols	Each member	Number of points	Frequency	Comparison between members, in search of the differences/similaritties and key characteristics of the family members
		Quality of points	Count positive and negative elements		Presence-absence, repetition	Family	Number of points	Frequency	Richness/poverty of contents and themes	Quality of points	Frequentcy of positive and negative elements	Quality of the themes/events and avoidance/repetition
							Presence-absence, repetition	Quality of the themes/events and avoidance/repetition
	Lines/connections	Each member	Number of lines	Frequency	Comparison between members, in search of the differences/similaritties and key characteristics of the family members
			Quality of lines (positive, negative, so-so)	Frequency of positive, negatives and so-so lines	
				Presence-absence, repetition	
Family		Number of lines	Frequency of positive, negatives and so-so lines	Comparison between positive and negative relatiosnhips
		Which points/symbols are connected and which aren’t	Observation of the points connected amomg them	Capability of giving value to the people/events within the family and/or outside the family
Center of the circle	Center of the circle	Occupied/empty	Observation of the center	Presence/absence of an element organizing the family
		Who/what occupies the center	Observation of who/what occupies the center	Power dynamics, roles played in terms of family organzation, influence and family relations
Border	Border of the circle	Crossed/not crossed by lines	Observation of the lines crossing the border	Openness toward the external environment on the side of one or more family members; assumption of a centriguge or centripetal position on the side of one or more family members
		Presence of points/symbols	Observation of the points/symbols on the border	Elements/themes occupying a marginal posotion with repsect to the family and the social context
Gestalt	Each member’s geometrical figure	Idetifying the figure connecting the points drawn by each family member	Observation and drawing of an imaginary line connecting the outer points of each member’s drawing in order to define the portion of space occupied by each family member	Comparison between the figures obatained for each family member in order to explore their closeness/dustance as well as their influence on the family structure and organization
	Family’s geometrical figure	Idetifying the figure connecting the points drawn by all the family members	Observation and drawing of an imaginary line connecting the outer points of the overall drawing in order to define the portion of space occupied by the family as a whole	Identifucation of the family form by comparing the family polygon with the circle: concentration, filling-saturation, measurement, separation
Comparison between the two administrations (present-future or present-past)	Each member’s drawing	Highlight the differences/similarities with respect to drawings by each family member	For each family member, identify the changes in the points, connections, positions with respect to the center and the border	Highlight the individuals’ willingness/openness to change (if the future versioni s administered); explore how each family member perceived the changes (if the past version is administered)
	Family’s drawing	Highlight the differences/similarities between the two drawings	Compare the two drawings overall in terms of the changes in the points, connections, positions with respect to the center and the border	Highlight the family’s openness to change (if the future version is administered); explore the perceived changes (if the past version is administered)

It should be reminded that the drawing interpretation is not solely based on individual and collective graphic productions; transcripts of family interactions and exchanges are also taken into consideration. Throughout the process, family exchanges contribute to understanding the qualitative attributes and meanings attached to each symbol, their positioning, and their mutual relations. The process of interaction in drawing is read qualitatively through the following indicators: cooperation (designing the drawing together and agreeing on who draws what), consensus (expressing verbal agreement on a member’s drawing), abstention (not interacting with family members during drawing or not drawing when prompted by a family member), dissent (verbally expressing objections or disagreements regarding someone’s drawing), conflict (drawing together becomes an occasion for argument or conflict among family members). The indicators have been selected from the most recent literature on observing family exchanges (Kerig and Lindahl, 2001; Seikkula et al., 2012). In this perspective, this instrument relies on a multidisciplinary approach.

The Family Life Space (FLS) enables clinicians to thoroughly and systematically analyze the essential elements present in the graphic-symbolic representation created by the family. The analysis of the areas and elements described above yields a wealth of information. However, these measurements do not automatically correspond to specific profiles or characteristics of family functioning. They require interpretation in alignment with the tool’s underlying theoretical assumptions and the unique attributes of the individuals within the family. For example, similar drawings in terms of portions of the space occupied by members or the position of the elements may have completely different interpretations depending on whether the family comprises only adults or also includes children. The same principle applies when considering the overall figure: the geometric figures resulting from connecting various elements in the drawing take on different meanings according to the elements involved in the figure formation (e.g., a parent or a child, a present or absent person, an organization, or a critical event).

For these reasons, it’s crucial to discuss the findings of the drawing with the family. This is a second and indispensable level of analysis that validates the hypotheses generated by the tool and unveils the underlying meanings of the geometric and spatial shapes produced. It also serves as a means for eliciting thoughts, emotions, memories, and plans. In this way, the tool becomes an opportunity for stimulating reflection and change.

## 5 Case study

A case study illustrating the use of the FLS as a family assessment tool will now be presented. The marital couple was referred to the psychologists by the family Court amidst a highly contentious separation process. The family is composed of the mother, father and three siblings, two of which are adults and have long started living independently.

We will first examine the drawing created by the family and then proceed with a clinical interpretation of the results using the indicators outlined above. The instrument was administered to a family consisting of a father (53 years old), a mother (45 years old), and their son, Pietro (14 years old).

**Figure 1:**
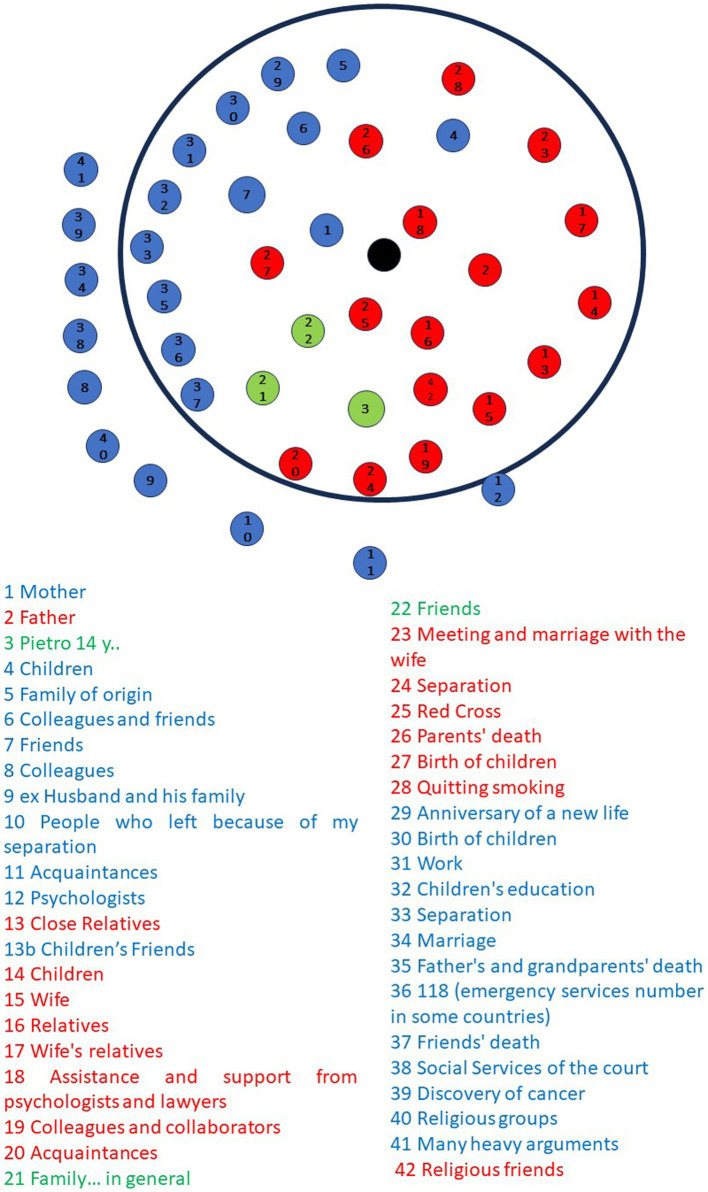
Graphic-symbolic drawing made by the family: points.

Figure 1 displays the family’s drawing. It is immediately evident that the family, comprising three present members, engaged in the assigned task in a quantitatively uneven and unbalanced manner, both in terms of the number of symbols and connections.

Regarding the elements in the drawing, the mother (indicated by the color blue) drew 23 points, the father (red) drew 15 points, and Pietro (green) only drew two elements. While the parents positioned themselves in the middle of the circle, symmetrically with respect to the center (see points 1, 2, 3), Pietro placed himself below and in an intermediate position between them. There is a clear prevalence of points drawn by the mother, while Pietro’s perspective is underrepresented.

In terms of content, the mother, in addition to family members and her job, depicted her extended family network, significant positive and negative life events, both personal and family-related, and the people connected to them. The father, in addition to family members, represented his colleagues as well as some personal and family life events. Pietro represented the family with a single point and subsequently used another point to collectively represent his friends. Regarding the quality of the elements in the drawing, both parents depicted positive and negative events. However, Pietro did not represent any aspect of his life, except, as previously mentioned, his family and friends. There are multiple repetitions between the two parents, especially regarding family members, work colleagues, and some specific events (i.d., the birth of their children, their marriage, and separation).

As shown in Figure 1, the mother starts drawing and is followed by the father and their son. After the first round, the mother and father take turns drawing various elements.”

**Figure 2:**
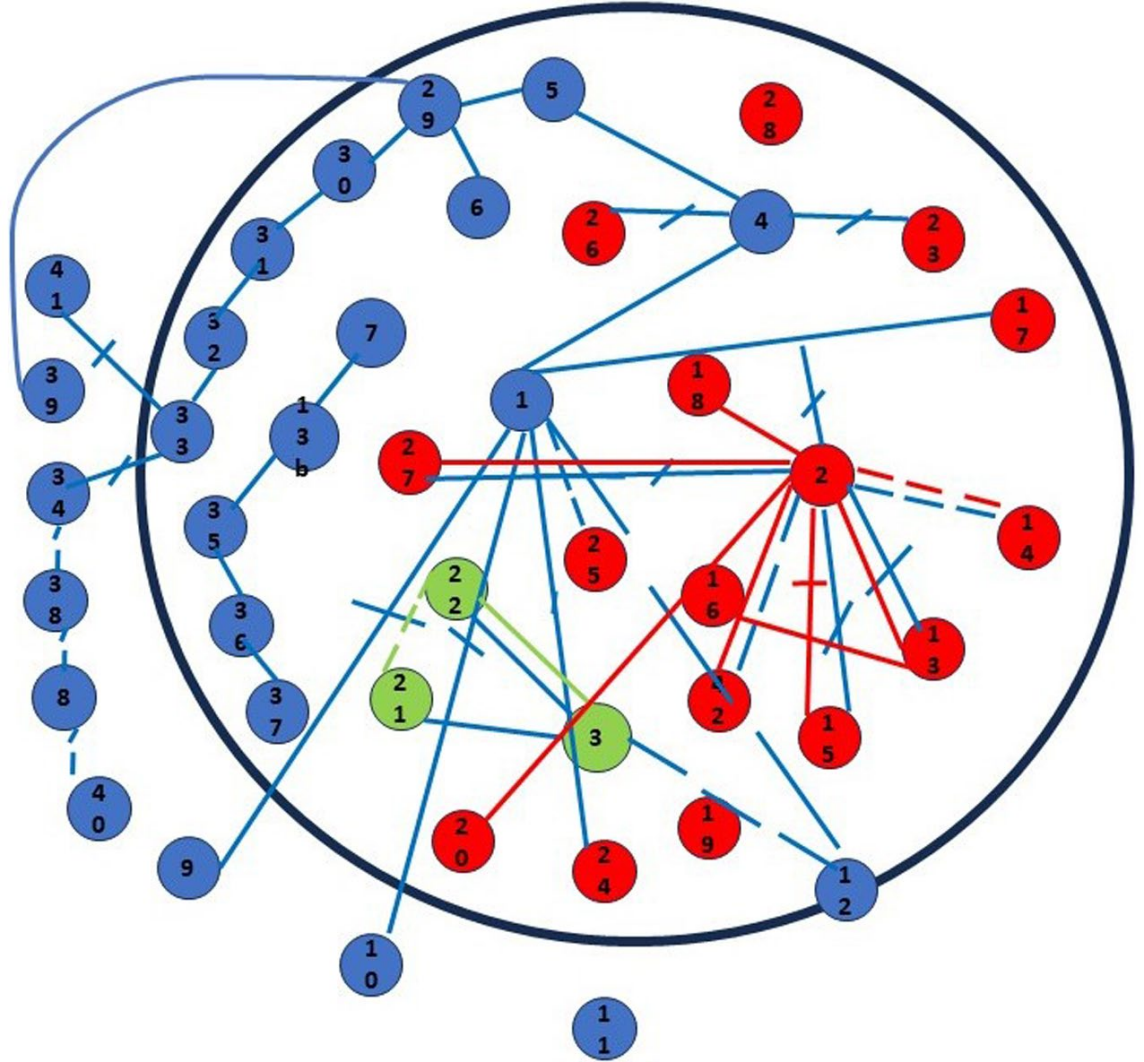
Graphic-symbolic drawing made by the family: connections.

Several conclusions can be drawn by examining the connections between the elements (see Figure 2): the mother not only links the points she has drawn among themselves but also traces lines connecting her son’s and husband’s points. She eventually connects some of her points with those of her husband, totaling 34 relationships drawn by the mother. The father draws nine connecting lines exclusively among the symbols he drew, while the son draws only two lines, solely among his symbols. Once again, the mother represents more relationships and is the only one connecting the symbols of the other family members.

Concerning the quality of the mother’s relationships, 16 are considered positive (i.e., represented by continuous lines) and tie her symbols and those drawn by her son, 10 are seen as negative (i.e., an interrupted line) and mainly regard her husband’s symbols and some specific events in her life, eight are viewed as fair (i.e., represented by a dotted line) and they are evenly distributed among her own symbols and those of her husband. In conclusion, positive relationships are drawn between elements of the mother’s personal life and her family members, while problematic or conflictual relationships are observed regarding her marriage, separation, and some members of her husband’s family. Relationships with the institutions and other professionals involved in the separation are ambivalent (“so-so”). Particularly interesting is the observation of the negative relationships drawn by the mother, including between the father and the children, the father and some relatives of him, the father and her, the father and the birth of their children, the children and the death of paternal grandparents and the marriage of the parents. Additionally, negative relationships involve the husband and her relatives, and the relationship between the mother and the separation.

The father positively connects himself to the birth of their children, himself to religious groups as well as to acquaintances and close relatives. Negative relations he drew with his former wife while the bond with their children is seen as ambivalent. The only connection that does not directly involve him is the positive relationship between the relatives and close relatives.

Pietro links his points by drawing an ambivalent relationship between his family and his friends and a positive relationship between himself and his friends.

When looking at the process, it should be noted that the parents took turns and almost shadowed one another when asked to draw connections between the points. Pietro remained on the sidelines until the parents had completed their work, and then he drew his two relationships.

With regards to the center of the circumference, Figure 2 shows it is occupied by two lines. The mother’s line indicates a conflictual relationship, while the father’s line is indicative of a positive one, both the lines connect the father to the birth of the children.

On the other hand, the boundary of the circumference is crossed by five lines (four of which indicate conflicting relationships), drawn by the mother. The border is also occupied by a point, once again drawn by the mother, representing the psychologists. The outside of the circumference is occupied by the mother with nine points representing events and people with whom she has a conflictual or ambivalent relationship. This includes her marriage, illness, some people she considers hostile as well as her husband and his family.

**Figure 3:**
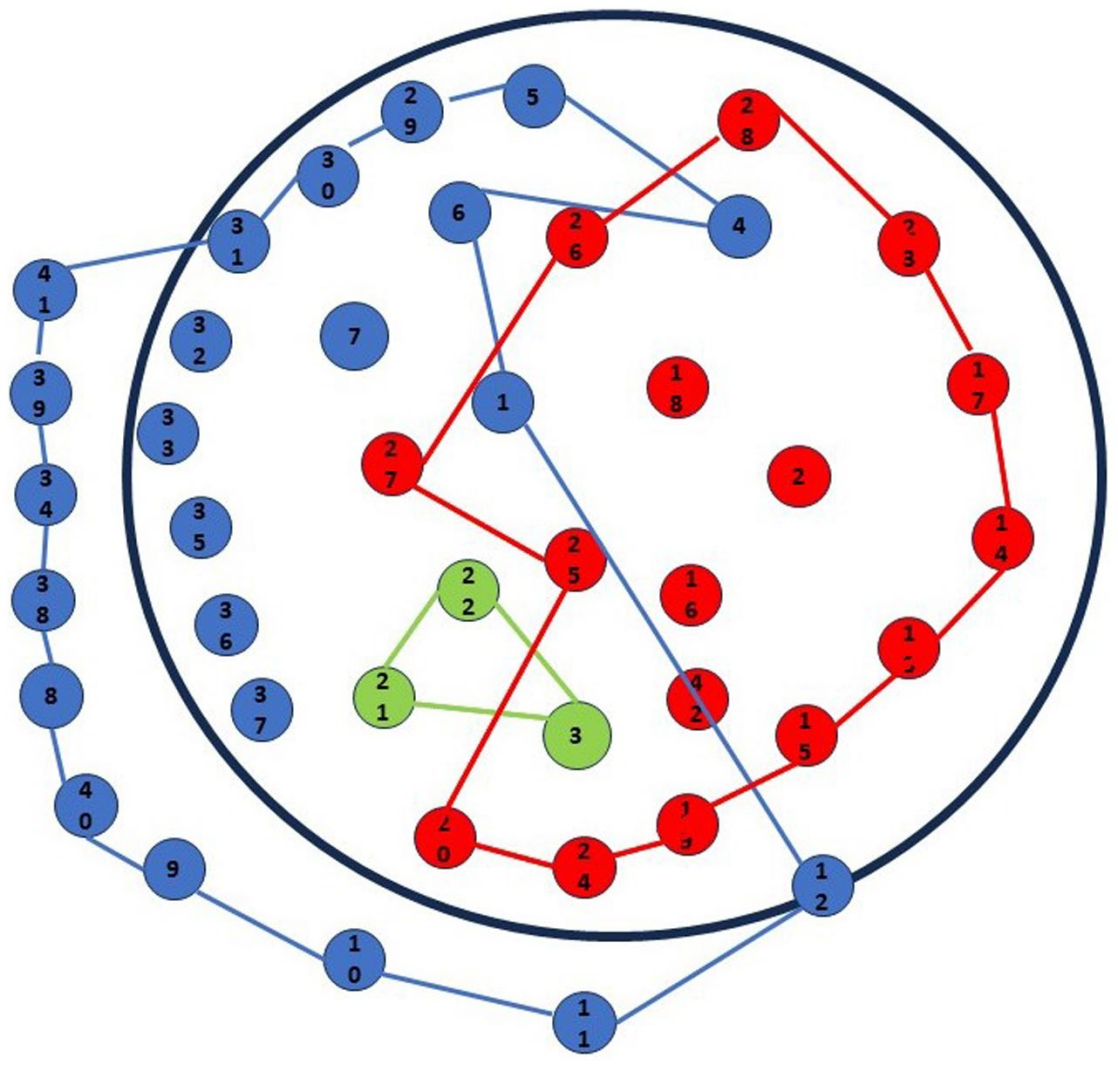
Graphic-symbolic drawing made by the family: individual and family shapes.

Now, the overall gestalt of the drawing will be considered (see Figure 3).

The points drawn by the mother and father occupy two opposing and symmetrical areas of the sheet with extensive overlapping in the middle. The balance and tension between the maternal and paternal realms, encompassing the entire circle, are clearly visible. In contrast, Pietro’s drawing occupies a relatively small area on the sheet, primarily within the mother’s domain and partially within the father’s.

The polygon formed by connecting the outermost points in the family drawing mirrors the shape of the circumference but also extends beyond its border. The circumference is entirely filled with points.

**Figure 4:**
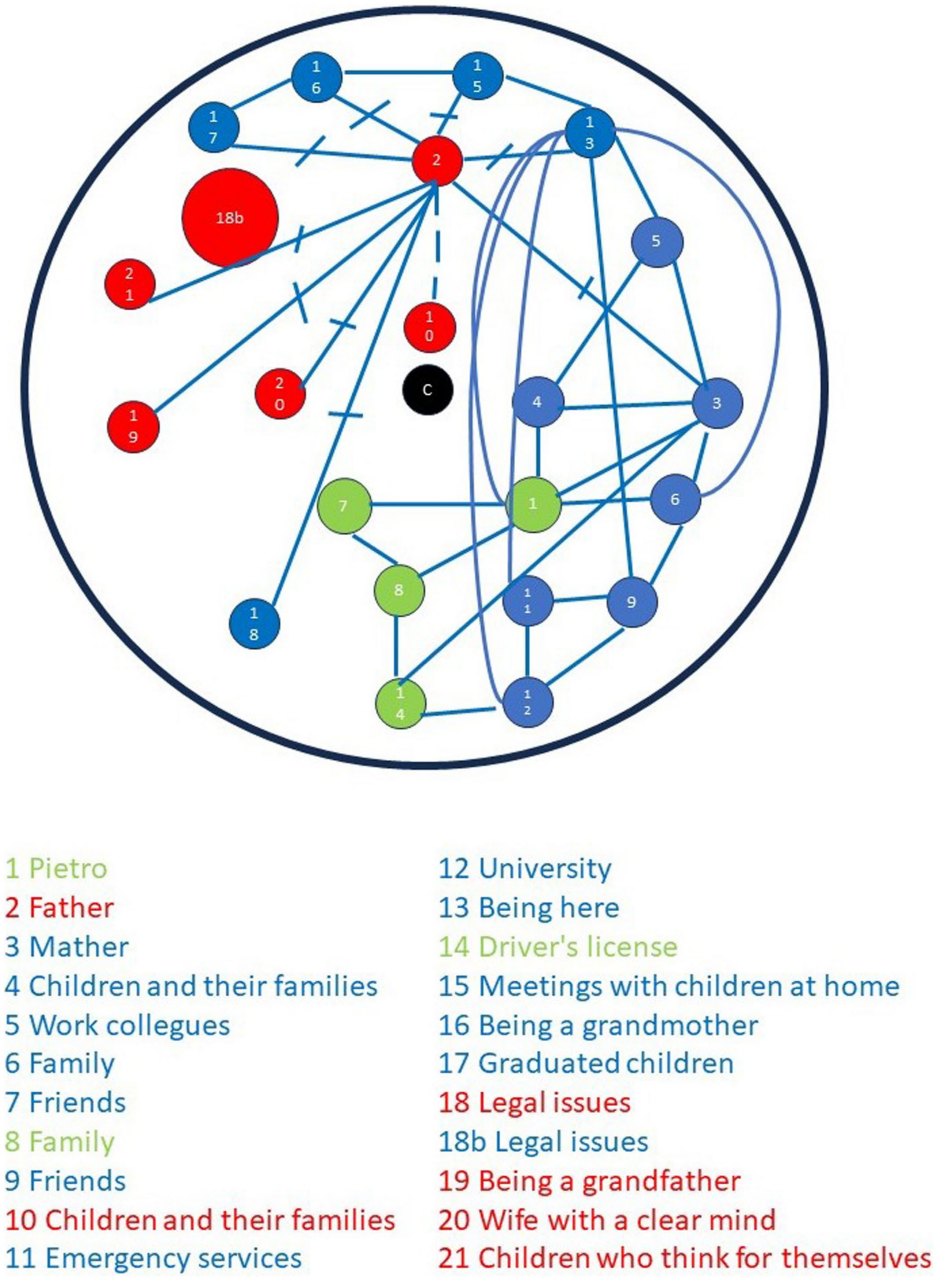
Graphic-symbolic drawing made by the family: the future (in 5 years).

In addition to the present version, Pietro’s family also completed a future iteration of the FLS. Specifically, the family was asked to envision their situation 5 years from the present. Figure 4 depicts the results of this second administration.

In terms of the points, the mother draws 11, the father draws six, and Pietro draws four. Notably, Pietro initiates the drawing process, followed by his father and then his mother. The events depicted are predominantly positive, except for a symbol drawn by both parents representing legal issues. The interactive and fluid alternation of family members on the sheet during this second administration is evident in the legend of Figure 4.

The relationships between points are only drawn by the mother and Pietro. The mother connects her points positively, as well as those of her son. Conversely, all relationships between her points and those drawn by her former husband are either negative or ambivalent. Pietro establishes positive connections between his points, envisioning positive relationships with his family and friends.

The center is now vacant, with a symbol representing the future families of the children just above it. The circumference’s boundary, as well as the area outside the circle, are not occupied by any symbols of lines.

**Figure 5:**
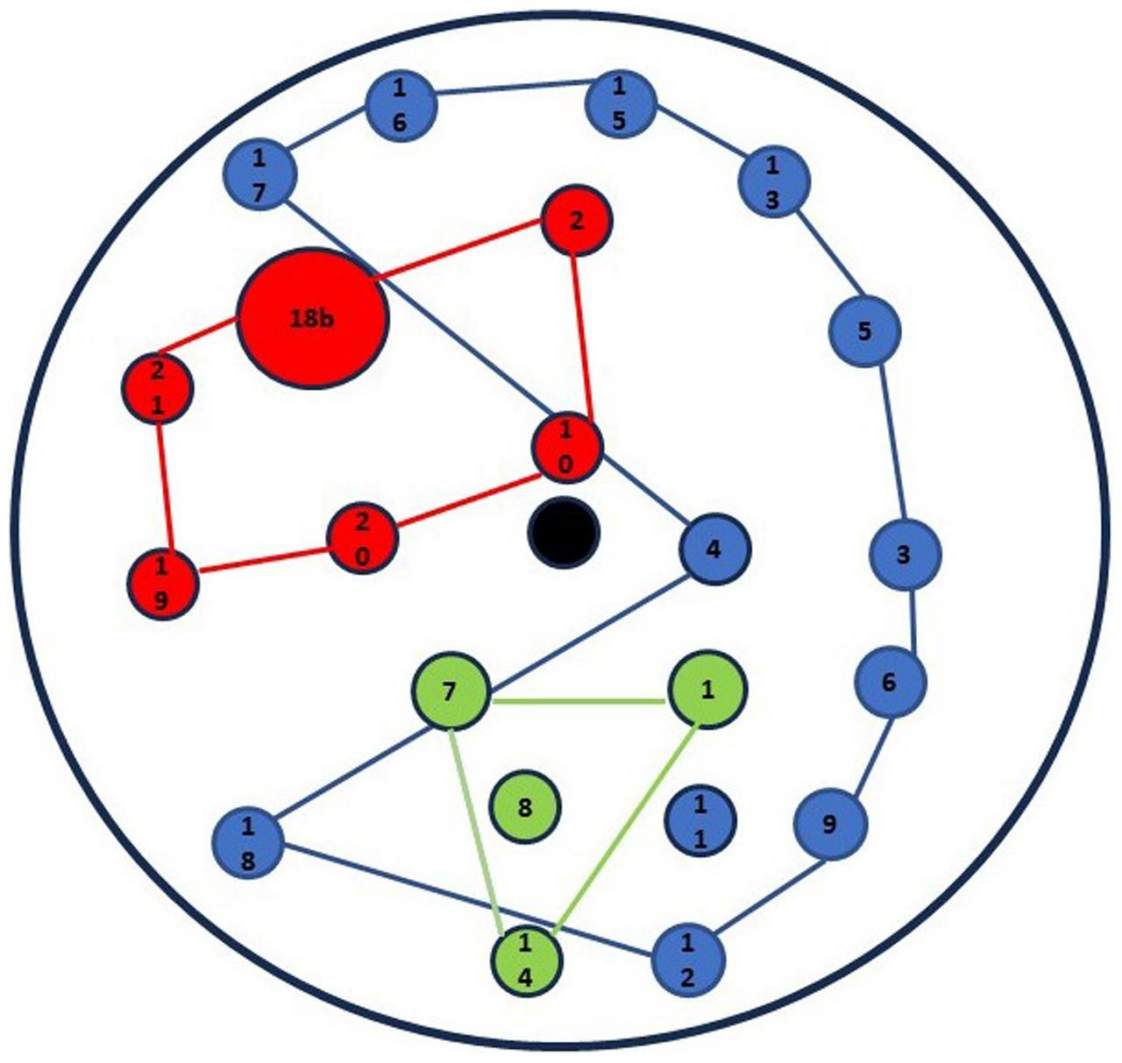
Graphic-symbolic drawing made by the family: individual and family shapes in the future (in 5 years).

Regarding the area occupied by individual representations, Figure 5 reveals a contraction of the areas occupied by the parents and a slight expansion of Pietro’s area. Specifically, the mother’s area remains the largest, while the father’s area is considerably more limited. There is a partial overlap between these two areas, and Pietro’s area is almost entirely enclosed in the mother’s domain.

The figure obtained by connecting all the family members’ points fills the circle but white space can still be seen: the points and relationships do not appear to completely saturate the area inside the circumference. This configuration can be classified a measured, according to the definitions provided above. Measurement is characterized by each family member occupying a specific sector of the circle while symbols and relationships are well differentiated (see Figure 2).

The comparison between the present (see Figure 2) and future (see Figure 4) versions of the FLS shows some interesting changes. Firstly, the parents reduce the number of points they each draw, while Pietro’s presence is increased, albeit only with the addition of only one element. In terms of quality, the points drawn by both parents lose their negative connotation: only legal issues are viewed as negative and drawn by both parents. It is interesting to note that Pietro adds the “driver’s license” as a significant event. The father does not draw any relationships in the second administration, while the mother’s only problematic relationships are those related to her former husband; all the relationships with her own as well as with Pietro’s symbols are positive. Pietro confirms his positive connections with his friends and family. In the second version, the center is no longer occupied, and the boundaries and outside area appear empty. When the area occupied by each family member is observed, a less poignant contraposition between the mother’s and father’s drawings is noted: while the father shrinks his domain both in terms of number of points and connections, the mother still takes up a large amount of space with both points and connections between them.

In both the administrations the mother takes up a domineering role, to the point that she is the one drawing the larger number of elements and connections, moreover, a difficult relationship with the area outside the circle can be observed both in the present as well as in the future version. While in the present the points falling outside the circle are extremely close to its border and are generally given a negative connotation, in the future version there are no points or lines outside of the circumference.

## 6 Clinical interpretations

The data and conclusions derived from administering the Family Life Space (FLS) contribute to formulating a clinical interpretation of the family. As previously mentioned, the FLS was administered to three individuals within the same family: Pietro, 14 years old; the father, 53 years old; and the mother, 45 years old. Pietro is the youngest of four siblings, with the other three being above 18 at the time of the evaluation.

Over the past 8 years, the parents have been entangled in a highly conflictual judicial separation, involving the family court. The father contested the judge’s decision on custody arrangements, claiming persistent difficulties in visiting his children. Specifically, he found it impossible to see his youngest son, Pietro, in the recent period. On Pietro’s part, he does not wish to adhere to the judge’s decision regarding visitation schedules.

Given this situation, the judge referred the entire family to a psychologist for an evaluation of family dynamics, aiming to better understand Pietro’s needs and decisions and assist in developing more functional relations between the adolescent and his parents. The evaluation process unfolded gradually, with individual sessions with the parents and children separately and some couple sessions before the FLS administration with the entire family.

What insights can we glean from the FLS administrations?

To begin with, it is readily apparent that the mother commands a substantial presence on the sheet: she is the one initiating the drawing. Moreover, her symbols outnumber those drawn by her ex-husband and son. The father draws fewer symbols, occupying a smaller opposing space to that of his former wife. Pietro, in contrast, depicts only two points in addition to himself and positions them beneath his parents’ symbols. The mother plays a major role in determining family dynamics, exerting power and influence over the other members, as we will later elaborate on with other indicators.

Regarding the content (quality of the elements in the drawing), Pietro’s representation is limited to the essential (his family and friends), while the mother’s drawing is detailed, depicting both positive and negative events (these latter are mainly related to her ex-husband, the separation, and illness).

The father’s drawing includes symbols representing his family and life events, most given a positive connotation, except for the separation. The elements provide a rich understanding of the parents’ lives, especially the profound impact of the separation. Pietro remains a passive observer of his parents’ drawing process.

Connections in the drawing confirm previous observations, with family patterns recurring across administrations: the mother explicitly conveys the negative connotation attached to her relationship with her former husband, the post-separation life, and the discovery of the tumor. Conversely, her other relationships, particularly those with her family and job are seen as positive. In this second administration, the mother goes to the point of connecting elements she did not draw (i.e., her ex-husband and her son). More specifically, the connections regarding Pietro are viewed as positive by the woman, whereas those pertaining her former husband are mainly negative. In this perspective, she imposes her interpretation on elements drawn by other family members, taking up all the space available and drawing a great number of lines. The father connects only the symbols he has drawn: positive (between himself and the birth of his children, himself and some religious groups, himself and acquaintances and close relatives), negative (between himself and his former wife), and ambivalent (himself and the children) connections are present. Thus, the father also expresses the conflict associated with the separation and the relationship with his ex-wife; in this perspective, his representation is both similar and opposed to that of his ex-wife. The drawing becomes the arena in which each of the parent re-enacts the family conflict in front of they son, Pietro, who observes impassively.

The mother and father have different views on specific relations; while they both consider father-children, father–mother, and father-close relatives’ relations as negative, the relationships between the father and religious groups and the father and the birth of the children are viewed negatively by the mother and positively by the father. Of particular interest is the divergent perception between the mother and father regarding the father’s relationship with the birth of their children. This connection assumes significant symbolic importance, and the mother disqualifies the father’s experiences at the birth of the children, including Pietro.

It’s crucial to note that Pietro, the youngest child, is present and exposed to his parents’ conflicting views during the administration.

In light of this data, we question whether the irreconcilable differences between the partners emerged after the birth of the children or if the contrasting view of the father-children relationship sparked the conflict.

Pietro draws only two connections, expressing positivity with his friends and challenges between his family and friends. While typical for an adolescent, this picture raises questions about the hindrances to a positive relationship between Pietro’s family and friends. These two domains currently remain irreconcilable, prompting further investigation by clinicians to shed light on Pietro’s world.

Regarding boundaries, the drawings by the father and son never extend beyond the circumference, signifying that life is only possible within its safe haven. The circumference, as per FLS instructions, represents family space. The outside holds no psychological relevance and is uninhabited; individuals can only envision themselves within the family. We question whether this space outside family life will ever be inhabited, considering Pietro’s developmental phase involves forming relationships and experiences outside the family.

From the father’s perspective, the impossibility of crossing the boundary is relevant, posing questions due to the pervasiveness of family conflict. The mother, on the contrary, can inhabit the space outside the circumference with points and lines crossing the boundary. Symbols and connections outside the circumference have negative connotations, portraying the outside as populated by threatening and painful aspects—an attempt to externalize or distance oneself from those difficulties.

From a Gestalt perspective (see Figure 3), the geometric figures formed by connecting the points of each family member confirm their positions and roles: the mother occupies the largest portion of space, and the figures of the mother and father complement each other, occupying opposing yet partially overlapping areas on the sheet. These contrasting positions suggest a conflict between the parents, involving various elements, including Pietro and the parents themselves. Pietro’s space is very limited, enclosed within his mother’s drawing and partially within his father’s. Consequently, Pietro is caught in the tensions between his parents that leaves him with no room for himself.

The hypothesis emerging from the Family Life Space (FLS) is that the intense conflict between the parents not only involves Pietro but also fails to provide the adolescent with sufficient space free from his parents’ interference.

With regards the first drawing concerning the present moment, the family gestalt can be classifies as a form of “filling-saturation.” The family space is dense and filled with points and relationships (see Figure 2), indicating limited possibilities for opening up to new events, as the family space within the circumference is entirely occupied. Interpreting such an indicator prognostically is complicated, as the current dysfunctional dynamics seem to hinder any change.

It is the comparison between the current and future version of the FLS that allows to draw some conclusions regarding the space for change available to this family.

In the future version, both the mother and father draw a lesser number of points, and these points mainly carry a positive connotation, except for the judicial separation. Pietro adds one element to those drawn in the present version: the driving license, symbolizing partial autonomy. The fact that Pietro initiates the drawing indicates greater participation and assertiveness. Moreover, when the drawings contents are considered, greater individual and family proactivity can be acknowledged.

With regards to the connections between the elements, the mother replicates the same patterns shown in the previous administration: she connects her own and Pietro’s symbols with straight lines, indicating positive relationships. The lines connecting herself to her ex-husbands’ symbols as well as those connecting the man’s points are, instead, dotted, to suggest an ambivalent, “so-so” relationship. Pietro’s connection are all positive while the father does not draw any connecting lines. The persistence and repetition of the same dysfunctional and invasive pattern on the mother’s side strike as problematic. On the other hand, the father appears to give up on relationships, while Pietro proposes a positive resolution to the present conflict. The family demonstrates the capacity for change, with the father and son being the main promoters.

Interestingly, the center of the circumference is now empty; just above the center, there is a point representing the children’s future families. This point might signify the family’s ability to evolve and change.

In the second version of the FLS, the border and the area outside the circumference are empty, indicating a persistent difficulty for the family in envisioning connections with the outside world, possibly due to intense internal conflicts.

Considering both individual drawings and the overall graphic production, Figure 5 reveals significant changes compared to the previous FLS. The parents now occupy a smaller portion of the space available, indicating a limited yet not precluded possibility to redefine the spaces occupied by each family member and the relationships among them. The overlap between the mother’s and father’s drawings is reduced, and Pietro’s polygon is now only partially enclosed within that of his mother.

The different drawing obtained in the future version suggests a more balanced distribution of space within the circumference and a reduction in conflict between the parents. From a gestalt point of view, the family’s drawing can be now classified as “measured”; according to the definitions provided above, measurement occurs when each family member occupies a specific sector of the circle and their symbols and connections are well-differentiated (see Figure 2).

## 7 Conclusion

A large body of research, documenting the impact of a family’s functioning on health outcomes, highlights the importance of introducing the evaluation of family dynamics into clinical judgment.

It’s abundantly clear that delving into the intricate dynamics of family life demands a nuanced approach like the multiple informant methodology proposed by Wagner et al., (2010). This methodological framework proves indispensable for capturing the nuanced interplay of interpersonal dynamics within families. By employing a diverse array of quantitative, qualitative, or mixed methods, researchers can delve deep into the multifaceted nature of familial relationships.

Quantitative methods, for instance, offer a structured means of extracting individual perceptions and experiences within the familial context. These data points can then be statistically transformed into dyadic scores, enabling researchers to glean insights from multiple perspectives within a family unit. Within scholarly literature, a plethora of quantitative scales exists, each stemming from a systemic understanding of family dynamics. These scales are designed to explore various family theoretical constructs such as cohesion, flexibility, communication, affectivity, commitment, and problem-solving (Hamilton and Carr, 2016).

However, it’s noteworthy that the Family Life Space (FLS) predominantly adopts a qualitative stance. Qualitative methods focus from mere quantification to a deeper exploration of the ‘how’ behind familial interactions, prioritizing the qualitative richness of shared experiences (Lanz et al., 2017). Thus, the qualitative approach offers a more holistic understanding of family dynamics, emphasizing the intricacies of relational dynamics over mere statistical metrics.

By encouraging collaborative data production among family members, qualitative approach acknowledges the family unit as a whole—a web of interdependent individuals rather than a mere sum of its parts. Morover, the term “family functioning” - from a family system perspective which assumes that the family members are part of a complex integrated system-refers to the ability of the family to work together as a unit to satisfy the basic needs of its members (Ryan and Keitner, 2009). Hence, it becomes imperative to utilize instruments that authentically evoke family interactions, enabling the observation of their dynamics within an ecological framework that minimizes deviations from real-life settings.

There is a scarcity of instruments in our repertoire designed to observe families in action and offer comprehensive insights into their dynamics. Among those familiar to us, we note Family Sculpture (Onnis et al., 1994), the Conjoint Family Drawing (Gennari and Tamanza, 2022), the Family Interaction Game (Favez et al., 2016), and the Lausanne Trilogue Play - LTP (Fivaz-Depeursinge and Corboz-Warnery, 1999), the Double Moon (Greco et al., 2020). The commonality among them lies in the systemic observation of family members engaging in activities, yet the specific observation indicators may vary. The FLS unquestionably falls within this category, sharing with the aforementioned tools not only a theoretical background but also the capacity to conduct clinical research while being applicable in clinical practice. Their utilization merges diagnostic assessment with prognostic aims, and the empirically significant information they yield complements clinical endeavors by stimulating facets of awareness and reflection.

The Family Life Space (FLS) holds numerous advantages, combining methodological robustness with adaptability across various contexts. It serves as an interactive tool for evaluating families from a relational perspective. Methodologically, the coding procedure relies on specific and objective elements, facilitating the collection of easily verifiable information that can be shared among clinicians and researchers (Mascolo, 2016). This instrument offers insights at individual, relational, and interactive levels, utilizing a unique approach to studying family relationships and providing specific insights into family dynamics. Unlike self-report instruments that gather individual perceptions, the FLS is a collaborative task involving all family members simultaneously. Self-report instruments are often ill-suited for investigating family relations, which are better assessed through interactive and relational tools (Seale, 1999; Tagliabue and Lanz, 2004). Interactive tools for generating relational information are currently rare and largely confined to clinical or qualitative use (Gilgun and Sussman, 2014).

Furthermore, the interactive nature of the FLS does not require family members to directly engage with the researcher while disconnecting from their family system. Instead, information is gathered by observing the family in its own environment, adopting an ecological perspective. The collaborative nature of the task allows each individual’s production to be viewed within a larger context and in relation to those of other family members.

As demonstrated in the case above, the set of elements identified and gathered through the instrument’s analysis serves as a valuable guide for the subsequent clinical interpretation of the family-provided information. Rather than offering a mere interpretation, it facilitates a shared, intersubjective understanding of the obtained information.

Moreover, its simple instructions and straightforward administration procedure make the instrument extremely versatile and easily applicable to both research and clinical assessment. In research settings, the FLS can compare different families, considering their respective lifecycle stages or the events members are facing (e.g., birth of a child, death of a parent, adolescence of a child, etc.). Comparisons can also be made regarding family structure and functions, such as parenting roles or the position held by children within the family or couple dynamics.

In clinical practice, the FLS allows for the interpretation of individual, relational (dyadic), and gestalt aspects of the family. Considering all these intertwined aspects provides insight into family dynamics and allows for the emergence of a holistic, complete picture. Finally, its simple instructions and administration procedures make it suitable for various individuals and families, including those with limited language proficiency (Gennari et al., 2015).

## Data availability statement

The original contributions presented in the study are included in the article/supplementary material, further inquiries can be directed to the corresponding author.

## Ethics statement

Ethical approval was not required for the study involving humans in accordance with the local legislation and institutional requirements. The studies were conducted in accordance with the local legislation and institutional requirements. Written informed consent for research purposes was obtained from all adult participants engaged in the clinical assessment. Written informed consent for participation in this study was provided by the participants’ legal guardians/next of kin. Written informed consent was obtained from the individual(s) for the publication of any potentially identifiable images or data included in this article.

## Author contributions

MG: Writing – review & editing, Writing – original draft, Methodology, Investigation, Conceptualization. CG: Writing – review & editing, Writing – original draft, Methodology, Investigation, Conceptualization. GT: Writing – review & editing, Writing – original draft, Methodology, Investigation, Conceptualization.
